# Medication use and the risk of motor vehicle collision in West Virginia drivers 65 years of age and older: a case-crossover study

**DOI:** 10.1186/s13104-016-1974-x

**Published:** 2016-03-15

**Authors:** Toni M. Rudisill, Motao Zhu, Danielle Davidov, D. Leann Long, Usha Sambamoorthi, Marie Abate, Vincent Delagarza

**Affiliations:** Department of Epidemiology, West Virginia University, PO BOX 9151, Morgantown, WV 26506 USA; Injury Control Research Center, West Virginia University, PO BOX 9151, Morgantown, WV 26506 USA; Departments of Emergency Medicine and Social and Behavioral Sciences, West Virginia University, PO BOX 9151, Morgantown, WV 26506 USA; Department of Biostatistics, West Virginia University, PO BOX 9151, Morgantown, WV 26506 USA; Department of Pharmaceutical Systems and Policy, West Virginia University, PO BOX 9510, Morgantown, WV 26506 USA; School of Pharmacy, West Virginia University, PO BOX 9520, Morgantown, WV USA; School of Medicine, West Virginia University, PO BOX 8059, Morgantown, WV 26506 USA

**Keywords:** Prescription drugs, Nonprescription drugs, Automobile driving, Aged adult, Risk, Traffic accidents

## Abstract

**Background:**

The current generation of older adults reports a higher lifetime prevalence of prescription, over-the-counter, and recreational drug use. The purpose of this analysis is to characterize the drug usage and determine the risk of motor vehicle collision associated with individual medications in a population of drivers ≥65 years.

**Methods:**

A case-crossover study was conducted at West Virginia University Healthcare’s facilities using data obtained from the electronic health records (n = 611) of drivers ≥65 years admitted for medical treatment following a motor vehicle collision which occurred between Jan. 1, 2009 and June 30, 2014. Patients’ medication usage 14 days before collision were matched and compared to their medication usage during four control periods prior to collision. Odds ratios were then calculated for the most prevalent individual medications and pharmaceutical sub-classes using conditional logistic regression.

**Results:**

Analgesic, cardiovascular and gastrointestinal medicines were common. Few drivers tested positive for either licit or illicit drugs. Of those testing positive for drugs, benzodiazepines and opiates were prevalent. Drivers consuming Tramadol (adjusted OR 11.41; 95 % CI 1.27, 102.15) were at a significantly increased risk of motor vehicle collision.

**Conclusions:**

Older adult drivers who have a prescription for this medication may need to be aware of the potential risk. Further research is necessary in a larger, more nationally representative population.

## Background

The United States (US) is experiencing an unprecedented demographic shift as the number of adults 65 years of age and older (i.e. seniors) are the most rapidly growing subgroup of the population [1]. By 2020, it is estimated that the number of licensed drivers over 65 years of age will exceed 40 million [2]. This demographic shift poses a unique challenge to both public health and traffic safety officials as senior drivers experience more motor vehicle fatalities [3] and an increased rate of injurious crashes per mile driven [4].

It is also well-established in the literature that medication usage increases with age. In the US, adults over 65 years of age consume more than 30 % of all annual written prescriptions [5]. The current generation of older adults reports a higher prevalence of both lifetime legal and illegal drug use compared to previous generations [6]. As more adults continue to drive later in life compared to previous generations, concerns regarding how these drugs affect driving ability are beginning to amass [7, 8]. While alcohol is a known contributor of motor vehicle collisions, the extent to which drugs other than alcohol contribute to crashes is less lucid [9]. There is evidence that driving under the influence of drugs is increasing nationally and that commonly prescribed prescription medications, some of which may interfere with safe driving, are becoming more ubiquitous than traditional illegal drugs among fatality injured drivers [10, 11].

The association between medications and motor vehicle collisions is largely understudied in the US [7, 8], particularly among senior drivers [12]. This issue may be of particular relevance to residents of West Virginia. The population of West Virginia is more mature compared to other states where ~16 % of the state’s residents are over 65 years of age [13] compared to ~13 % in the US population [14]. Previous research has also shown that both legal and illegal drug use contribute greatly to motor vehicle collisions in West Virginia [9]. The traffic fatality rate is also starkly higher—nearly 45 % more—compared to other non-Appalachian states [15]. Therefore, the purpose of this analysis is to explore which prescription and over-the-counter medications are most common, if illegal drug use is prevalent, and which prescription and over-the-counter medications are associated with an increased risk of motor vehicle collision among West Virginia drivers 65 years of age or older. While the intent of drug use is often impossible to determine, prescription and over-the-counter-medications will be referred to as ‘licit drugs’ in this analysis because it shall be assumed that these substances were taken to remedy a medical condition and obtained from a legal facility; ‘illicit drugs’ will refer to traditional illegal drugs, such as methamphetamine, cocaine, etc., which are often obtained illegally, abused, and have no real medical benefit. Discerning this information may help guide future interventional and educational efforts to minimize death and disability from motor vehicle collisions in this population.

## Methods

### Study design

The design used for this analysis was a case-crossover. Developed by Maclure in 1991, case-crossover studies are similar to matched case–control studies in theory; the fundamental difference between the two study designs is that cases serve as their own controls in a case-crossover study [16]. The case-crossover design compares (i.e. matches) a case’s exposure during a time period immediately preceding an event (i.e. the case or ‘risk’ period) to the exposure in a time period when the event did not occur (i.e. control period) [16]. These designs are useful for studying the relationship between transient exposures and acute outcomes; they are commonly used in air pollution studies, traffic safety, and pharmacoepidemiology [17]. A benefit of the case-crossover design is that fixed confounders, such as age, race, sex, etc., are controlled for regardless if they were actually measured [18].

**Fig. 1:**
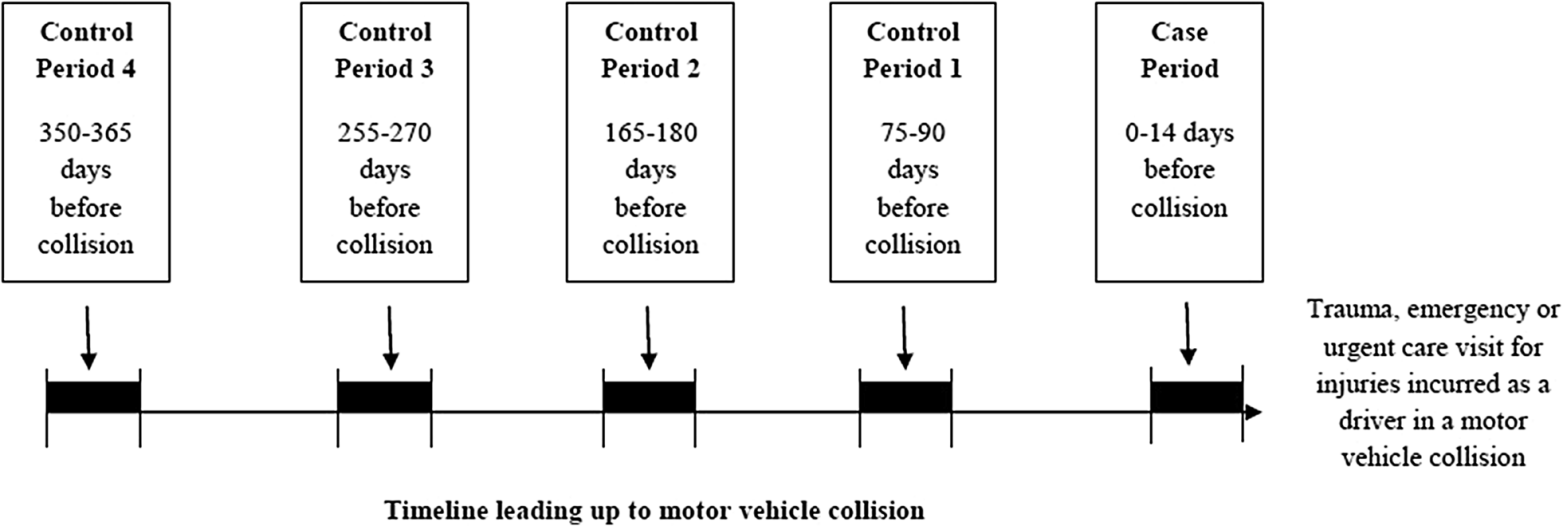
Overview of sampling schema for case and control periods

The sampling schema used for this analysis is depicted in Fig. 1. The event of interest was a motor vehicle collision in which the driver required medical treatment. The exposure of interest was the driver’s medication usage leading up to the collision. The risk period was defined as the 14 days preceding the motor vehicle collision. The reason as to why 14 days was chosen as the risk period as opposed to a shorter duration was because medications may take time to accumulate and/or cause side-effects in an individual which may interfere with their driving ability. Additionally, this length of risk period has been used in previous studies [19]. In order to increase statistical efficiency [20], there were four matched control periods, each 15 days long, at 350–365, 255–270, 165–180, and 75–90 days before the collision. Control periods were chosen to be disjoint from one another to avoid possible correlation [21].

### Study setting

All cases received medical treatment from West Virginia University (WVU) Healthcare’s facilities located in Morgantown, WV. Morgantown is a city with approximately 29,600 residents situated in Monongalia county in north-central West Virginia [22]. The size of the town can fluctuate throughout the year as it is home of West Virginia University, the state’s largest institution of higher education. In 2012, approximately 25.1 % of the patients served by WVU Healthcare resided in Monongalia county, while an additional 21.5 % of patients lived in surrounding counties (i.e. Preston, Marion, and Taylor counties) [23]. Approximately 38 % of patients who sought treatment from WVU Healthcare were from other West Virginia counties, while another ~15 % of patients were from surrounding states (i.e. Pennsylvania, Maryland, Ohio, Virginia and Kentucky) [23]. Therefore, the population served by WVU Healthcare is fairly representative of the state. The population of West Virginia is predominately of white non-Hispanic race/ethnicity and is comprised largely of older individuals [22]. In 2010, heart disease, cancer, and chronic lower respiratory disease were the leading causes of death in this population [22].

### Data sources and collection

All data for this analysis were collected from the electronic medical records of eligible cases and obtained through Medsite and Epic (Merlin), WVU Healthcare’s electronic medical record systems. Data was collected through a combination of manual data abstraction and through the assistance of the West Virginia University Clinical and Translational Science Institute’s (WVCTSI) Integrated Data Repository. In order to ensure the congruence of the data obtained from WVCTSI and the actual medical records, several electronic records were pulled and compared to ensure accuracy. This study was approved by West Virginia University’s Institutional Review Board (protocol #1401165743).

### Case selection

The sampling frame consisted of individuals 65 years of age and older at time of treatment and received treatment from emergency or trauma services or urgent care facilities from January 1, 2009 to June 30, 2014. Cases had to be designated as a driver involved in a motor vehicle collision by the International Classification of Disease, Ninth Revision, Clinical Modification codes (ICD 9-CM) E810.0, E811.0, E812.0, E813.0, E814.0, E815.0, E816.0 and E819.0. The E-codes used to identify the incident cases could have been listed as the primary or secondary diagnosis in the electronic medical record.

### Medication exposures

Medications of interest were those considered potentially driver-impairing, meaning that they could possibly alter a driver’s cognition, psychomotor function, or physical functioning as suggested by the American Medical Association [24]. Medication usage up to a year before each case’s admittance for treatment was assessed. Medications that were started up to 24 h before admittance for treatment were not included in the analysis; this was done to avoid potential bias in the event that a medication was administered to a patient by emergency medical services (i.e. the medication was consumed after the collision occurred). The duration of time that an individual was taking a particular medication was obtained directly from the medical record. If the duration of medication usage could not be determined or was not documented in the record, the medication was not included in the analysis as to avoid misclassification. As many of the cases were existing patients of WVU Healthcare and/or due to the severity of the incurred injuries, medication records were fair in terms of completeness. Of the 611 case visits included in this analysis, 581 (95 %) cases had medications noted in their electronic health record at any point, while 292 (48 %) cases had medication usage in the year preceding their motor vehicle collision. Of these individuals, 286 (47 %) were taking a medication during the risk/case period. An individual was considered ‘exposed’ if they were taking a medication at any point during the case and/or individual control periods.

While the purpose of this analysis was to explore the risk of motor vehicle collision due to individual medication exposures, an analysis of more encompassing pharmaceutical sub-classes was also conducted. This included anticholesteremics, anticoagulants, antidepressants (non-benzodiazepines), antihyperglycemics, antihypertensives, narcotic analgesics, and benzodiazepines. In this particular analysis, anticholesteremics included 3-hydroxy-3-methyl-glutaryl-CoA reductase inhibitors. Anticoagulants included both coumarin type and platelet aggregation inhibitors. Antidepressants included tricyclic antidepressants, selective serotonin re-uptake inhibitors, serotonin antagonist and reuptake inhibitors, serotonin–norepinephrine reuptake inhibitors, norepinephrine-dopamine reuptake inhibitors, and noradrenergic and specific serotonergic antidepressants. Antihyperglycemics included insulin, biguanides, insulin-release stimulants, combination biguanides and insulin-release stimulants, and thiazolidinediones. Antihypertensives included angiotensin-converting-enzyme inhibitors, angiotensin receptor antagonists, and sympatholytic vasodilators. Narcotic analgesics also included narcotic-non salicylate combinations, and anesthetic adjunct agents.

### Other covariates

Other variables of interest, which could help describe the population, included age, gender, race, day, time, year, and season of admittance, how the patient was transported to medical treatment, treatment location, injury severity, insurance status of the patient, county and state of residence, employment status, length of hospital stay, and health status. The categorization of these variables is presented in Table 1. For season of admittance, winter included December, January, and February, while spring comprised March, April, and May. Summer included June, July, and August while fall included September, October, and November. Treatment location was grouped into trauma, emergency or urgent care departments; because so few patients were seen by urgent care (i.e. less than 10), urgent care and emergency were reported together to protect patient confidentiality. Patient’s injury severity was based on acuity level at time of arrival for treatment. Patient acuity levels that were noted as emergent or immediate were classified as severe injuries. Acuity levels that were urgent were classified as moderate injuries. Acuities that were less urgent or non-urgent were classified as minor injury severity. Since acuity is not assigned within the Urgent Care facility, these were left unassigned. Insurance status of the patient was grouped as government, private, none, or other/unknown. Government insurance included Medicare or Medicaid programs. Private insurance indicated that a patient had a commercial insurance plan. Employment/work status was categorized as retired or employed. If a patient reported that they were still working full, part-time or were self-employed, employment status was categorized as employed. Chronic disease status was categorized as 0, 1–3, or 4 or more based on the number of chronic conditions patients were noted to have in their medical records. For patient confidentiality purposes, these conditions were grouped as opposed to listing each individually. The chronic conditions of interest, along with Clinical Classification Software codes or ICD 9-CM codes used to identify them in patients’ records, were as follows: heart disease (96, 97, 100, 101, 103–108, 114, 117), stroke (109, 111, 112, 113), dementia (653), Alzheimer’s disease (331.0), diabetes (49,50), cancer (11–44), arthritis (201–203), Parkinson’s disease (79), hypertension (98–99), asthma (128), chronic obstructive pulmonary disease (127), alcoholism (660), depression (296.2, 296.3, 311), anxiety (651), chronic kidney disease (158), and substance abuse/dependency (661). As part of the admission process, patient’s urine may have been laboratory tested for the presence of alcohol and/or drugs. The drugs that could be detected were amphetamines, barbiturates, benzodiazepines, buprenorphine, cannabis, cocaine, opiates, phencyclidine and propoxyphene. Results of drug testing were also noted (Table 3).

**Table 1:** Demographic characteristics of patients

Characteristic	Patient visits (N = 611)	
	N	(%)
Age (years)		
65–69	225	(36.8)
70–79	252	(41.2)
80–89	119	(19.5)
≥90	15	(2.5)
Gender		
Male	330	(54.0)
Female	281	(46.0)
Race		
White	560	(97.0)
Other	17	(3.0)
Missing	34	
Day of admittance		
Mon–thurs	368	(60.2)
Fri–sun	243	(39.8)
Time of admittance		
7:00 AM–6:59 PM	462	(75.7)
7:00 PM–6:59 AM	148	(24.3)
Season of admittance		
Winter	133	(21.8)
Spring	189	(30.9)
Summer	147	(24.1)
Fall	142	(23.2)
Year of admittance		
2009	102	(16.7)
2010	87	(14.2)
2011	89	(14.6)
2012	154	(25.2)
2013	110	(18.0)
2014	69	(11.3)
Method of transport		
Ambulance	248	(40.6)
Helicopter	62	(10.2)
Self	37	(6.1)
Other/unknown	264	(43.2)
Treatment location		
Trauma	135	(22.1)
Emergency and urgent care	476	(77.9)
Injury severity		
Minor	29	(9.0)
Moderate	43	(13.4)
Severe	250	(77.6)
Unknown	289	
Insurance status		
Government	331	(54.2)
Private	181	(29.6)
None	30	(4.9)
Other/unknown	69	(11.3)
County of residence		
Monongalia	92	(15.4)
Other	506	(84.6)
Missing	13	
State of residence		
WV	491	(80.4)
Other/missing	120	(19.6)
Work status		
Employed	74	(12.4)
Retired	523	(87.6)
Missing	14	
Number of chronic conditions		
0	108	(17.6)
1–3	314	(51.4)
≥4	189	(31.0)
Average length of stay (days ± SD)	1.9 ± 4.9	

*SD* standard deviation

### Statistical methods

Because of the 1:4 matching of case to control periods, conditional logistic regression was used to calculate the odds of motor vehicle collision for each medication exposure [25]. Because of the study design (i.e. known longitudinal medication exposure over time), the odds ratio approximated the incidence rate ratio (i.e. risk) [26]. To account for the slight difference in exposure lengths of case and control periods (i.e. 14 versus 15 days, respectively), the natural log of exposure time in days was used as the variable offset as suggested by Greenland [25]. Because case-crossover studies are not immune to within person confounding, all regression models were adjusted for the number of medications (both prescription and over-the-counter) a case was taking during each risk and control period. The number of medications used served as a proxy of health status as a case’s health may have been time-varying (i.e. improved or declined) over the study period. Analyses could not be adjusted for the number of chronic conditions a case possessed as this did not change over the study period. All analyses were run using SAS/STAT Software version 9.3 [27], with α = 0.05.

## Results

The demographic characteristics of cases and the circumstances surrounding their medical visits (n = 611) are presented in Table 1. The majority of cases were aged 65–69 (36.8 %) or 70–79 years (41.2 %) at time of treatment. More males (54.0 %) than females (46.0 %) were admitted for treatment post-collision and most were of white race (97.0 %). As less than 10 patients were seen at urgent care, most patients were treated in the emergency department as opposed to trauma service (22.1 %), though many of the injuries sustained were moderate to severe. Most patients had government (54.2 %) or private (29.6 %) insurance coverage. The majority of patients were West Virginia residents (80.4 %). While most patients were no longer working, 12.4 % still held some form of employment. Chronic conditions (i.e. 1 or more) were common (82.4 %).

Analgesic, cardiovascular and gastrointestinal medications were the most prominent therapeutic groups observed during case and control periods (Table 2). As for specific medications, Aspirin, Metoprolol, Lisinopril, and Furosemide were the most common. A combination of Oxycodone and Acetaminophen was the most common dual-drug compound.

**Table 2:** Most frequently identified medications during case and control periods

Total cases (N = 611)	Number and percentage of cases taking these drugs N (%)	
Broad therapeutic groups		
*Analgesics*	82	(13.4)
*Cardiovascular*	81	(13.3)
*Gastrointestinal*	74	(12.1)
*Psychotherapeutics*	48	(7.9)
Specific medications		
*Metoprolol*	38	(6.2)
*Aspirin*	33	(5.4)
*Esomeprazole*	30	(4.9)
*Lisinopril*	27	(4.4)
*Furosemide*	21	(3.4)
Combinations		
*Oxycodone and Acetaminophen*	20	(3.3)

Only 32 % of patients were tested for drugs at time of admittance, while slightly more were tested for alcohol (Table 3). Overall, traditionally illicit drugs were not detected in cases. Among those testing positive, benzodiazepines and opiates were the most detected substances. Approximately, 50 % of individuals testing positive for opiates or benzodiazepines had a traceable prescription for these substances in the past year before collision.

**Table 3:** Alcohol and/or drugs identified in cases via laboratory testing at time of admittance

Substance	Cases tested N (%)	Cases testing positive N (%)	Cases testing positive who had prescriptions for the identified drug N (%)
Alcohol	269 (44.0)	12 (4.5)	–
Drugs	194 (31.8)	61 (31.4)	–
*Benzodiazepines*	194 (31.8)	33 (17.0)	16 (48.5)
*Opiates*	194 (31.8)	30 (15.5)	15 (50.0)

Patients could be tested for the following drugs: amphetamines, barbiturates, benzodiazepines, buprenorphine, cannabis, cocaine, opiates, phencyclidine and propoxyphene. Benzodiazepines and opiates were the only drugs detected among patients tested

After adjusting for the number of medications (prescription and non-prescription) a driver was taking during each case and control period (Table 4), individuals (N = 11) who were taking Tramadol (OR = 11.41; 95 % CI 1.27, 102.15) were at a significantly increased risk of motor vehicle collision while taking this substance during the risk period compared to control periods. Though not statistically significant, those taking Clopidogrel, Gabapentin, Citalopram, Insulin, Hydrochlorothiazide, Metoprolol, Zolpidem, and Nitroglycerine were trending towards an increased risk of collision.

**Table 4:** The risk of involvement in a motor vehicle collision by medication exposure

Medication	Number of individuals taking medication (N)	1:4 Matched control periods^a^			
		Model 1 OR (95 % CI)		Model 2 OR (95 % CI)	
Anticholesteremic					
*Simvastatin*	12	1.00	(0.12, 8.17)	0.42	(0.03,07.23)
Anticoagulants					
*Clopidogrel*	10	10.73	(1.19, 96.67)	7.62	(0.48, 122.10)
*Warfarin*	10	0.72	(0.06, 9.04)	0.30	(0.08, 1.22)
Anticonvulsants					
*Gabapentin*	15	2.69	(0.59, 12.32)	1.32	(0.24, 7.17)
Antidepressants					
*Citalopram*	10	3.01	(0.31, 29.65)	3.21	(0.24, 42.50)
Antihyperglycemics					
*Insulin*	12	15.76	(1.78, 139.61)	2.63	(0.14, 48.72)
Antihypertensive					
*Furosemide*	12	0.50	(0.08, 3.22)	0.81	(0.06, 10.62)
*Hydrochlorothiazide*	13	12.35	(1.35, 113.06)	15.01	(0.76, 296.60)
*Lisinopril*	25	1.56	(0.43, 5.69)	0.27	(0.05, 1.60)
*Metoprolol*	29	5.29	(1.31, 21.38)	1.16	(0.23, 5.79)
Muscle relaxants					
*Albuterol*	11	0.44	(0.06, 3.20)	0.25	(0.02, 3.34)
Narcotic analgesics					
*Hydrocodone*	15	1.32	(0.36, 4.92)	0.37	(0.04, 3.79)
*Tramadol*	11	10.56	(1.17, 95.51)	11.41	(1.27, 102.15)
Sleep medications					
*Zolpidem*	10	4.20	(0.73, 24.13)	1.42	(0.66, 3.00)
Steroids					
*Fluticasone*	11	0.56	(0.08, 3.89)	0.41	(0.04, 4.85)
*Prednisone*	12	0.12	(0.01, 1.07)	0.19	(0.02, 1.82)
Vasodilators					
*Nitroglycerin*	12	2.54	(0.42, 15.23)	1.27	(0.07, 23.82)
Other drugs					
*Alendronate*	10	1.15	(0.22, 6.04)	0.13	(0.01, 1.78)
Combination drugs					
*Oxycodone and Acetaminophen*	16	0.61	(0.18, 2.00)	0.17	(0.02, 1.63)

^a^Conditional logistic regression was used to calculate the odds ratios and 95 % CI. Each case’s medication exposure during the 14 day risk period immediately before the crash was matched to four separate control periods up to 1 year before the collision to assess if medication use during the risk period was associated with an increase of motor vehicle collision compared to control periods. Model 1 is the crude estimate (i.e. unadjusted) while Model 2 was adjusted for the number of medications a person was taking during each case and control period

Table 5 lists larger therapeutic classes of medications along with subsequent crude and adjusted odds ratios and 95 % CI to approximate the risk of an individual’s involvement in a motor vehicle collision while taking these substances during the risk period compared to control periods. After adjusting for the number of medications a driver was taking during each case and control period, all therapeutic classes were not found statistically significant. Although those taking anticoagulants, antihyperglycemics, and antihypertensive medications during case periods were trending towards an increased risk of motor vehicle collision compared to control periods.

**Table 5:** Risk of involvement in a motor vehicle collision by medication exposure categorized by pharmaceutical sub-class

Medication sub-class	Number of individuals taking medication (N)	1:4 Matched control periods^a^			
		Model 1 Odds ratio (95 % CI)		Model 2 Odds ratio (95 % CI)	
Anticholesteremics	31	1.50	(0.44, 5.18)	0.42	(0.08, 2.21)
Anticoagulants	20	3.59	(0.84, 15.28)	2.29	(0.35, 15.19)
Antidepressants	30	2.05	(0.59, 7.16)	0.50	(0.09, 2.67)
Antihyperglycemics	22	15.36	(1.79, 132.0)	2.24	(0.17, 29.84)
Antihypertensives	39	3.32	(1.15, 9.62)	1.24	(0.29, 5.32)
Benzodiazepines	21	1.96	(0.58, 6.62)	0.71	(0.15, 3.34)
Narcotic Analgesics	41	1.56	(0.72, 3.39)	0.94	(0.32, 2.75)

^a^Conditional logistic regression was used to calculate the odds ratios and 95 % CI. Each case’s medication exposure during the 14 day risk period immediately before the crash was matched to four separate control periods up to one year before the collision to assess if medication use during the risk period was associated with an increase of motor vehicle collision compared to control periods. Model 1 is the crude estimate (i.e. unadjusted) while Model 2 was adjusted for the number of medications a person was taking during each case and control period

## Discussion

Two principal findings were generated as a result of this analysis. First, while few patients were tested for drugs at time of medical treatment, the drivers found drug-positive tended to test positive for common prescription medications. Typical illegal drugs, such as cocaine or phencyclidine, were not commonplace in this population. Second, despite small sample sizes, those taking Tramadol (N = 11) were at a significantly increased risk of motor vehicle collision if they took this substance 14 days prior to collision compared to control times. Numerous other medications were also trending towards an increased risk of motor vehicle collision, but were likely not found statistically significant due to small sample sizes and low statistical power. While it is possible that the medical conditions for which these drugs were prescribed may have influenced a patient’s driving ability, these findings may be of important clinical relevance and worthy of further exploration in a larger population.

Trend analyses have shown that prescription medications, particularly benzodiazepines and narcotic analgesics, are being detected more frequently than traditional illicit substances, such as cocaine and methamphetamine, among drug-positive drivers in the US [10, 11]. While the literature regarding drug usage among older adult drivers is limited, previous research has suggested that illicit drug usage may not be common among this population. A study of drug usage among level one trauma patients over 60 years of age involved in motor vehicle collisions (n = 180) in Tennessee during the 1990’s revealed that alcohol and illicit drugs were detected in only 14 and 1 %, respectively, of study participants [28]. Therefore, the findings from this analysis were similar. The current analysis also showed that approximately 50 % of patients had a traceable prescription for benzodiazepine or opiates within the year before collision. It is possible that the other 50 % of drivers received a prescription outside of WVU Healthcare network, consumed pills left over from older prescriptions, possibly from sharing medications, obtained them illegally, or simply had incomplete documentation in their medical records. This finding may be worthy of additional exploration.

As for the risk of motor vehicle collision posed by individual medications, two previous studies have investigated the risk of motor vehicle collision associated with the use of Tramadol [29, 30]. In the study by Bachs et al., Tramadol use was not significantly associated with an increased risk of motor vehicle collision among a Norwegian cohort of adult drivers aged 18–70 years, though it was trending in the direction of increased risk (Risk 1.5; 95 % CI 0.9, 2.3). In the study by Gibson et al., Tramadol was significantly associated with an increased risk of collision (Risk 9.17; 95 % CI 7.81, 10.77) in an English cohort of drivers aged 18–74 years. At high doses, Tramadol is also known to affect balance [31]. Poor balance has also been linked to an increased risk of motor vehicle collision, particularly in older populations [32]. Therefore, the effects of Tramadol are likely not age-dependent because this analysis was limited to older adult drivers whereas other studies investigated drivers less than 74 years of age and the results were comparable.

Numerous other medications in this study were also trending towards an increased risk of motor vehicle collision, but were not found statistically significant included: Clopidogrel, Gabapentin, Citalopram, Insulin, Hydrochlorothiazide, Metoprolol, Zolpidem, and Nitroglycerin. To the authors’ knowledge, no studies have investigated whether Clopidogrel, an anticoagulant, affects driving ability. Two studies have investigated the relationship between anticoagulants as a pharmaceutical sub-class and the risk/odds of motor vehicle collision. A case–control study conducted by McGwin et al. showed that elderly drivers in Alabama during 1996 who consumed anticoagulants were 2.6 times more likely to be involved in a motor vehicle collision (OR 2.6; 95 % CI 1.0, 6.7) [1]. As to which medications were included in this categorization was not described. A case–control study conducted by Delaney et al. in Quebec, Canada showed that those taking Warfarin, another anticoagulant, were not at an increased risk of motor vehicle collision (OR 0.74; 95 % CI 0.55, 1.05) [33], which was similar to what was seen in this analysis. Gabapentin, a newer anti-epileptic medication, is not known to affect driving ability, though it may. In a study conducted by Martin et al., healthy senior adults (mean age 66.5 years) experienced mild cognitive effects during psychomotor testing conducted in a laboratory setting [34]. Common complaints regarding Gabapentin use include dizziness, nausea, and somnolence [35], which could affect one’s ability to drive. While Citalopram has not been associated with an increased risk of motor vehicle collision in two other studies [30, 36], second-generation antidepressants as a pharmaceutical sub-class are known to effect driving ability possibly due to side effects produced after initial use [37]. It is possible that those taking Citalopram in this analysis were experiencing side-effects, though this is unknown. Several studies have investigated the association between Insulin use and the risk of motor vehicle collision [1, 38–42]. Most of the findings concerning Insulin use are mixed. Two studies have found significantly increased risks of motor vehicle collision with Insulin use [38, 40], while four others have found no significant associations [1, 39, 41, 42]. To the authors’ knowledge, no studies have investigated the risk of motor vehicle collision associated with individual antihypertensive medications. One study by McGwin et al. did investigate the use of angiotensin-converting enzyme (ACE) inhibitors, beta-blockers, and diuretics, which all can be used to treat hypertension [1]. After adjusting for age, sex, race, and annual miles driven, the odds of motor vehicle collision were 1.6 (95 % CI 1.0, 2.7), 1.4 (95 % CI 0.8, 2.3), and 0.9 (95 % CI 0.5, 1.7) for ACE inhibitors, beta blockers, and diuretics, respectively [1]. In this analysis, Hydrochlorothiazide, a diuretic, and Metoprolol, a beta blocker, were both trending toward being associated with an increased risk of motor vehicle collision. While the pharmacokinetic properties of these drugs may be a potential explanation, the reason as to why the effects of Hydrochlorothiazide on motor vehicle collision were so pronounced is unknown. Zolpidem, a sleep-promoting medication, has consistently shown in three other studies to be associated with an increased risk of motor vehicle collision [32, 43, 44]. While no studies have investigated the effects of individual vasodilators, such as Nitroglycerin, on motor vehicle collision, only one study investigated them as a pharmaceutical sub-class. Mcgwin et al. found that vasodilators as a sub-class were not associated with an increased risk of collision [1]. The reason as to why Nitroglycerin use in this analysis was trending towards an increased risk of motor vehicle collision remains unknown.

The findings from this analysis have several key clinical implications. While these results are not conclusive, it may be worthy of notifying older patients who have prescriptions for these medications of their potential risk, particularly if they drive frequently. Future interventional efforts could involve raising patient awareness. Secondly, the findings of this study may suggest that drugs within the same pharmaceutical class maybe more or less driver impairing than others. This may be worthy of consideration to clinicians when prescribing medications to patients who drive frequently.

The findings from this analysis do need to be interpreted with caution as disease-medication relationships are often difficult, if not impossible, to distinguish. It is entirely possible that the disease in which the medications were prescribed could be affecting one’s ability to drive. Numerous medical conditions have been associated with motor vehicle collision, particularly sleep apnea [45], dementia [46], arthritis [47], diabetes [48], epilepsy [48], anxiety [49], depression [49], and Parkinson’s disease [50]. In addition, drugs that affect the central nervous system may exhibit different effects among individuals. There are numerous intrinsic and extrinsic factors that can alter medication effectiveness and/or side effects among individuals. These include, but are not limited to, drug solubility [51], intestinal pH [51], drug interactions [52], age [53], sex [53, 54], weight [53], diet [55, 56], genetics [57], circadian rhythms [58], supplement use [59], health of the individual [60], developed tolerance [61], dosage [62], route of administration [62], etc.

Several of these factors which may alter a drug’s effectiveness were controlled for by study design; the strength case-crossover studies are that all fixed confounders are controlled. Despite this strength, this study has several distinct limitations. First, certain time-varying covariates were not adjusted for as many of them were immeasurable. For example, driving exposure (i.e. the amount that an individual drives), is a known and important confounder of traffic studies [63] and could not be accounted for in this analysis because it is absent from the medical records. Second, there were limitations associated with the medical records. Much of the information collected was self-reported by the patient or their legal guardian and subject to recall bias. Some of the information, such as medication duration, may have been incomplete or was simply not documented. Also, the accuracy of E-codes for identifying potential cases in this analysis may have been lacking [64]; therefore some potential cases may have been unnecessarily excluded particularly if the patient was not coded properly. Third, patient behavior was unknown; it was impossible to determine if the patient was taking their medication as prescribed by their healthcare provider. Fourth, the sample sizes in this analysis were often small and many statistical tests were likely under-powered. Also, several regression analyses were run, so statistical significance could have been achieved by chance alone. Fifth, as this study was conducted in only one state, the findings may not be generalizable to other locations. Sixth, as previously mentioned, disease-medication relationships are often difficult to distinguish. The findings of this analysis are not suggesting that the medications were the cause of the motor vehicle collision; the findings are associative. Future research may involve replicating this study in a larger population that is nationally representative.

## Conclusion

This analysis sought to characterize drug usage and determine which individual medications were associated with an increased risk of motor vehicle collision among West Virginia drivers 65 years of age or older using a case-crossover approach. It was determined that analgesics, cardiovascular and gastrointestinal drugs were prevalent in this population. While few drivers were tested, illicit drug use was uncommon. Those taking frequently prescribed medications, such as Tramadol, were at a significantly increased risk of motor vehicle collision while taking these substances during the 14 day risk period compared to control periods. Future research in this area is necessary as different medications may pose more risk to patient safety than others. The association between medication use and the risk of motor vehicle collisions is particularly important considering that drivers are living longer and maintaining their mobility later in life despite their medical conditions.

## Abbreviations

CI: confidence interval
OR: odds ratio
SD: standard deviation
US: United States
WV: West Virginia
WVCTSI: West Virginia University Clinical and Translational Science Institute
WVU: West Virginia University
